# Development of an independent MU calculation software for radiotherapy treatments with stereotactic cones

**DOI:** 10.1002/acm2.13542

**Published:** 2022-02-15

**Authors:** Guilherme Filipe Pinto Campos, Ana Catarina Santos Souto, Joana Borges Lencart, Luís Paulo Teixeira Cunha, Anabela Gregório Dias

**Affiliations:** ^1^ Department of Physics and Astronomy Faculty of Sciences University of Porto Porto Portugal; ^2^ Medical Physics Radiobiology and Radiation Protection Group IPO Porto Research Centre (CI‐IPOP) Portuguese Oncology Institute of Porto (IPO Porto) Porto Portugal; ^3^ Medical Physics Department Portuguese Oncology Institute of Porto (IPO Porto) Porto Portugal

**Keywords:** heterogeneity corrections, independent MU calculation, SRS treatments, stereotactic cones, TRS‐483

## Abstract

**Purpose:**

Development of an independent MU calculator (StereoCalc) with and without heterogeneity corrections for stereotactic treatments, in a Varian TrueBeam STx LINAC using stereotactic cones, with flattening filter‐free photon energies.

**Methods:**

Multiple depth curves and output factors were measured, following the dosimetry formalism for small fields proposed by the TRS‐483. The developed StereoCalc imports and processes the beam data files and calculates the patient plans with and without heterogeneity correction. Validation of the developed software was carried out using phantoms. The accuracy of the StereoCalc software was verified in stereotactic patient plans.

**Results:**

A maximum difference of 2.47% and 2.07% was obtained in the phantom validation tests with and without heterogeneity correction, respectively. The mean percentual difference of StereoCalc from cone dose calculation (CDC) in the clinical testing was 2.86% ±1.27% and 0.78% ±0.48% with and without heterogeneity correction, respectively. The largest differences found were 7.34% and 1.98%, respectively.

**Conclusions:**

The results obtained in this work show that the MU calculated with StereoCalc software is in good agreement with the values calculated by the treatment planning systems, both in static fields and arcs. We have also improved the software to consider heterogeneity corrections calculations. As expected, and as a major achievement of this work, some differences were observed when heterogeneities were considered. StereoCalc proved to be a powerful tool that can be integrated into the specific quality assurance program in a medical physics department for independent verification in stereotactic treatment with cones.

## 1 INTRODUCTION

Stereotactic radiotherapy techniques allow the treatment of small lesions in the brain with high accuracy. The technique selection depends on the tumor size and its proximity to critical structures. According to the International Commission on Radiation Units and Measurements recommendation, a maximum dose difference of 5% between the planned dose and the delivered dose is accepted to ensure that the treatment is safe and effective.^1^ Sources of error might have origin in the linear accelerator (LINAC) commissioning, contouring, dose calculation, patient positioning, beam stability (flatness, symmetry, output, dose rate), or mechanical faults that can affect the LINAC performance, such as MLC, gantry, and collimator accuracy.

Although technology has reduced the risk of under or overexposure to radiation, many incidents were reported over the years due to the incorrect use of treatment planning systems (TPSs).^2^ To ensure the maximum achievable accuracy of stereotactic treatments, an exhaustive quality assurance (QA) program must be pursued.^3^, ^4^, ^5^ Furthermore, several international guidelines recommend an independent verification of the computer calculation output with a secondary calculation method.^1^, ^6^, ^7^

StereoCalc, an independent monitor unit calculator (MUC) for stereotactic treatments using stereotactic cones was developed in MATLAB to fulfill international recommendations such as the American Association of Physicists in Medicine (AAPM) TG‐114.^1^ This MUC is based on the dose calculation model of the cone dose calculation (CDC) algorithm from Varian, at the isocenter, for 6 MV and 10 MV flattening filter‐free (FFF) beams, to check the TPS calculated MU.

To ensure that the StereoCalc is truly independent, a set of relative measurements including multiple depth dose curves and output factors (OFs) were obtained, following the new dosimetry formalism proposed by the International Atomic Energy Agency TRS‐483: dosimetry of small static fields used in external beam radiotherapy.^8^, ^9^ This is the first international code of practice dedicated to the dosimetry of small fields for specialized radiotherapy equipment able to produce small fields and for conventional LINACs with and without flattening filter for photon fields up to 10 MV.

StereoCalc takes into consideration tissue heterogeneities, overcoming the main limitation of the CDC algorithm used in radiosurgery treatment dose calculations. For an efficient QA process, StereoCalc imports the DICOM plan and calculates the MU automatically based on the LINAC commissioning data such as the relative measurements and the absolute dosimetry measurement in reference conditions. StereoCalc as an independent MU calculator allows the prevention of major errors in dose delivery.

## 2 METHODS AND MATERIALS

### 2.1 Beam data acquisition

CDC is classified as a 1D calculation algorithm and belongs to the broad‐beam methods of dose calculation. It represents the dose of simple beams (conic collimators) using beam generating functions (analytical approach) based on the measured data. StereoCalc, on the other hand, represents the dose using tabulated beam data that are stored in tabular form, which is interpolated during dose calculation. Both calculation methodologies rely on the introduced beam data. To ensure that the MU calculator is truly independent, beam data required for the StereoCalc configuration were acquired and carried out according to the TRS‐483 for relative dosimetry of small fields in a Varian TrueBeam STx LINAC using 6 MV FFF and 10 MV FFF energies. All cones bundled with the LINAC were considered in this work: 4, 5, 7.5, 10, 12.5, 15, and 17.5 mm.

Tissue maximum ratio (TMR) was acquired with a PTW 60019 microDiamond detector in a PTW MP3 water tank system using a PTW Tandem electrometer. Measurements ranged from the water surface up to 20 cm depth. For each cone, the detector was positioned and centered on the radiation beam using in‐plane and cross‐plane scans to yield the maximum signal intensity. The synthetic diamond detector was positioned parallel to the beam central axis as recommended in TRS‐483. To ensure that the beam is parallel to the vertical axis of the water tank, a center check was performed for both axis at two different depths (5 and 20 cm) every time a cone was changed. TMR curves were interpolated to 1‐mm spacing and smoothed in PTW MEPHYSTO mc^2^ suite software. No reference detector was used during measurements due to the small cone sizes. To minimize the noise of the profile curves, the acquisition time and the delay time between each measurement were increased, and the detector moving speed was decreased. The dose rate was the same used clinically for radiosurgery treatments: 1400 MU/min for 6 MV FFF and 2400 MU/min for 10 MV FFF. TMR was normalized to the depth of maximum dose: 15 mm for 6 MV FFF and 25 mm for 10 MV FFF.

The OFs were acquired with a PTW 60019 microDiamond detector in a PTW MP3 water tank system using a PTW UNIDOS E electrometer. Each measurement was performed at 95‐cm SSD for 5 cm depth with 100 MU. The readings were normalized to the reference field of 10 × 10 cm^2^ using the same setup conditions. The same dose rate of TMR measurements was used.

TRS‐483 presents several tables with OF correction factors depending on the machine, energy, and detector used, as a function of the equivalent square field size (s). For circular small fields, as the cones used, the equivalent square field for each cone was determined using the Equation 1:

(1) $$s\;=\;r\times \sqrt{\pi }$$

where s is the half of the full width at half maximum (FWHM) diameter of the cone.

Field output correction factors were then fitted into a two‐term exponential curve:

(2) $$k_{Q_{\mathrm{clin}},Q_{\mathrm{ref}}}^{f_{\mathrm{clin}},f_{\mathrm{ref}}}\;\left(s\right)=\;a\times e^{b\;\times \;s}+c\times e^{d\;\times \;s}$$

where s is the equivalent square field size, and a, b, c and d are the parameters to be fitted. Parameters were found with the MATLAB fit function with an *R* ^2^ of 0.9998.

For PTW 60019 microDiamond detector, TRS‐483 protocol only specifies correction factors down to 4 × 4 mm^2^ equivalent square field. The smallest cone has a nominal diameter of 4 mm, corresponding to a 3.54 × 3.54 mm^2^ equivalent square field. To overcome this limitation, extrapolation was used for the smallest cone, using the same two‐term exponential curve of Equation 2.

### 2.2 Software development

StereoCalc consists of two stand‐alone applications: one to import and process the LINAC calibration data (*Beam Configuration*) and another to import, process, and calculate the number of MU from a DICOM plan (*Stereotactic MU calculator*). StereoCalc was developed in MATLAB R2019a from MathWorks. Both graphical interfaces were designed in MATLAB App Designer.

#### 2.2.1 Beam configuration application

*Beam Configuration* was created to import and process beam data such as TMR (generated from PTW MEPHYSTO mc^2^ suite saved as mc^2^ file format) and OF measurements, along with the absolute dosimetry calibration data of the reference field. Beam profiles were not required because the MU verification is performed at the isocenter. Additionally, this application also imports the computed tomography (CT) calibration curve to establish a relationship between the CT number and the relative electron density (RED) of each voxel of the CT scan. The purpose of this application is to save all the raw input data into a processed MATLAB data file (MAT‐File) with all the above data to be used in the *Stereotactic MU Calculator*.

Each MAT‐File contains all the required beam data such as absolute dose calibration parameters and relative measurements (TMR, OF) that are mandatory to calculate the dose in a phantom or patient at the isocenter. A MAT‐File should be created for each LINAC and energy configured. Every time a change is made in the CDC algorithm that affects beam data, absolute dose calibration, or CT calibration curve, a new MAT‐File should be created with the new data.

#### 2.2.2 Stereotactic MU calculator application

This application was created to calculate the number of MU based on a DICOM plan, which needs to be exported from the Eclipse TPS in DICOM format. The following DICOM objects are required to calculate dose: RT structure set, RT plan, RT dose, and the CT images of the patient or phantom. Dose calculation is performed based on the LINAC calibration data, which were imported and processed previously on the *Beam Configuration* application.

The dose on a single point inside a phantom or patient volume is calculated according to the following Equation 3:

(3) $$\begin{array}{ccc} & & D\;\left(r,d,SSD,S\right)=\;\text{MU}\times DR_{\mathrm{ref}}\times OF_{TMR_{\max}}\\ & & \;\left(S\right)\times \mathrm{TMR}\left(d,S\right)\times \left(\frac{\mathrm{SAD}}{\mathrm{SSD}+d}\right)\times \mathrm{OAR}\left(r,S\right) \end{array}$$

where D is the dose calculated on a single point with off‐axis distance r, depth d, source‐skin distance SSD with a cone with nominal size S and monitor units MU. $DR_{\mathrm{ref}}$ is the reference dose rate (ratio between the absolute dose in water for the reference field size at the reference point at the calibration depth and the number of MU given to produce the reference dose rate for calibration), $OF_{\mathrm{TM}R_{\max}}\left(S\right)$ is the OF at $\mathrm{TM}R_{\max}$, TMR is the tissue maximum ratio, SAD is the source‐axis distance and OAR is the off‐axis ratio. $OF_{\mathrm{TM}R_{\max}}\left(S\right)$ can be calculated with the Equation 4:

(4) $$\begin{array}{ccc} OF_{\mathrm{TM}R_{\max}}\;\left(S\right) & = & \;\text{OF}\left(S\right)\times \frac{\mathrm{PDD}\left(S,\mathrm{SSD},d_{\max}\right)}{\mathrm{PDD}\left(S,\mathrm{SSD},d\right)}\\ & & \times {\left(\frac{\mathrm{SSD}+d_{\max}}{\mathrm{SAD}}\right)}^{2} \end{array}$$

where $d_{\max}$ is the depth of maximum dose. Percentage depth dose (PDD) curves were derived from TMR curves. For more details about the conversion between TMR and PDD for small fields, the author is referred to the equation proposed by van Battum et al.^10^

Since the independent MU verification is carried out at the isocenter, there is no lateral shift, and there is no need to apply an inverse square law correction. It was assumed that OAR(r,S)=1 and $(\frac{\mathrm{SAD}}{\mathrm{SSD}+d})=\;1$.

Equation 5 to calculate the number of MU of a single field at the isocenter without heterogeneity correction is then:

(5) $$\mathrm{MU}\;=\frac{D\left(d,\mathrm{SSD},S\right)}{DR_{\mathrm{ref}}\times OF_{\mathrm{TM}R_{\max}}\left(S\right)\times \mathrm{TMR}\left(d,S\right)}\;$$

All the terms in Equation 5 are constants (such as $DR_{\mathrm{ref}}$) or are directly dependent on the reference conditions and cone size used (such as $OF_{\mathrm{TM}R_{\max}}\left(S\right)$). TMR(d,S) is the remaining term in the equation that needs to be determined and depends on two factors: the conical collimator diameter and depth. TMR calculation is straightforward on static fields as the depth is known and fixed. However, for arcs, once the depth varies as the gantry rotates, the average TMR needs to be calculated instead. Each arc is segmented in multiple incident gantry angles (with 1° resolution between each incident gantry angle) and for each gantry angle, depth is calculated to obtain the TMR value. An arc resolution of 1° was chosen for arc segmentation because it improves the accuracy of the average TMR calculation. Due to irregular surfaces such as the nose and the ears of patients, shorter arc resolutions may be beneficial.

Depth is calculated by tracing a straight line between the isocenter and the target of the LINAC and is given as the distance between the interception point on the patient surface along the central axis (CAX) and the isocenter. Its calculation depends on the isocenter location, gantry angle, couch angle, and the external contour. Depth is used for MU calculation without heterogeneity correction, and it is assumed that the patient body is homogeneously composed of water.

For MU calculation with heterogeneity correction, depth, d, was replaced by the effective depth, $d_{\mathrm{eff}}$, in TMR calculation in Equation 5. Effective depth or equivalent path length (EPL) is the distance in tissue weighted by the RED of that tissue to water, as shown in Equation 6. Being a method to correct heterogeneities inside the patient body such as bones or air cavities, this 1D inhomogeneity correction only works well for points that are far away from inhomogeneities. It does not represent the dose within the inhomogeneity because it does not consider the modifications of the scatter component, which has a greater influence within or close the inhomogeneity.^11^ This type of inhomogeneity correction is generally acceptable in the head region, since most beam paths are through approximately water equivalent densities, with a dose error of approximately 1.2% due to the skull attenuation.^12^

EPL is defined as:

(6) $$\mathrm{EPL}\;=d_{\mathrm{eff}}\;=\sum_{i}p_{i}^{el}\;\times x_{i}$$

where i represents each CT voxel along a given straight between the isocenter and the target, $p_{i}^{el}$ is the RED of voxel i, and $x_{i}$ is the voxel dimension along a given rayline. The RED of each voxel is retrieved from the CT calibration curve using the voxel Hounsfield unit value.

### 2.3 StereoCalc validation

MU calculation without heterogeneity correction was assessed by comparing the number of MU calculated with the StereoCalc and the CDC algorithm. Multiple plans were created in CDC using a homogeneous phantom (Lucy 3D QA) for both 6 MV FFF and 10 MV FFF energies with different conic collimator diameters (5 mm, 10 mm, and 17.5 mm), gantry and couch angles to reproduce different scenarios of irradiation both for static fields and arcs. Following AAPM TG‐114 formalism on the commissioning of independent MU calculators, a criterion of 2% dose difference was used for the validation of StereoCalc for fields with minimal shaping and using the same patient or phantom geometry.^1^

MU calculation with heterogeneity correction was validated using the QUASAR phantom from Modus Medical Devices. This phantom was chosen because it has multiple inserts with different densities and sizes, and the dose can be measured on the inserts or beyond them. Cedarwood insert was used to simulate zones of low density mimicking the thorax. These inserts were placed on both sides of the phantom, in the lung insertions. To simulate zones of high density, a bone insert was chosen. It was placed in the central bottom insertion of the phantom, in the vertebrae position.

A PTW 31016 PinPoint 3D was placed at the isocenter (center of the phantom) in a special insert designed for the detector. CT scan was acquired without the ionization chamber, but it was replaced by an insert with the same density as the ionization chamber holder.

**FIGURE 1:**
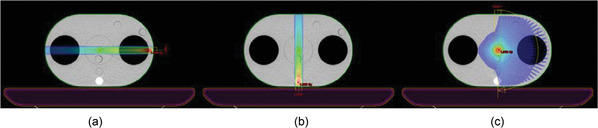
Static fields and arcs used to validate the MU calculation with heterogeneity correction using the QUASAR thorax phantom. (a) 90° static field, (b) 180° static field, (c) arc 0° → 180°

To study the dose attenuation in heterogeneous zones, multiple plans were created using CDC for 6 MV FFF and 10 MV FFF energies. A left lateral beam (crossing the lung insert – Figure 1a) and a posterior beam (crossing the bone insert – Figure 1b) were used. An additional arc crossing both the lung and the bone inserts during its trajectory (0° → 180° clockwise) was used to validate the MU calculation for arcs with heterogeneity correction (Figure 1c). Couch attenuation was taken into consideration in the calculation of the effective depth.

Due to the ionization chamber active volume and lack of output correction factors for fields below 10 × 10 mm^2^, only cones above 7.5 mm were used for this validation (10 mm, 12.5 mm, 15.0 mm, and 17.5 mm). The output correction factors used for the PTW 31016 PinPoint were obtained using the same methodology described for PTW 60019 microDiamond, fitting the field output correction factors into a two‐term exponential curve (Equation 2). An *R* ^2^ of 0.9997 was obtained for the calculated parameters of the curve.

### 2.4 StereoCalc clinical testing

To validate the accuracy of the developed algorithm, a total of 81 patients treated in a Varian TrueBeam STx using cones were chosen: 47 patients were planned with three arcs, 27 patients with four arcs, four patients with five arcs, and the remaining three patients were planned with 2, 6, and 8 arcs. A total of 285 fields were analyzed. All the treatment plans were generated with the CDC algorithm with 6 MV FFF photon beams. As mentioned before, this commercial algorithm does not consider heterogeneities. After treatment plan approval, the treatment plan data (RT Plan, RT Dose, RT Structure Set, and the CT images) were exported to StereoCalc.

The percentage differences between the MU calculated by CDC and the MU calculated by StereoCalc, with ($\mathrm{Dif}f_{\mathrm{WHC}})$ and without ($\mathrm{Dif}f_{\mathrm{NHC}})\;$heterogeneity corrections, were obtained using the following equations:

(7) $$\mathrm{Dif}f_{\mathrm{WHC}}\;\left(\%\right)=\;\left(MU_{\mathrm{WHC}}-MU_{\mathrm{CDC}}\right)/MU_{\mathrm{CDC}}\times \;100\;$$

(8) $$\mathrm{Dif}f_{\mathrm{NHC}}\;\left(\%\right)=\left(MU_{\mathrm{NHC}}-MU_{\mathrm{CDC}}\right)\;/MU_{\mathrm{CDC}}\times \;100\;$$

where $MU_{\mathrm{WHC}}$ and $MU_{\mathrm{NHC}}$ are the number of MU calculated by the developed algorithm with and without heterogeneity correction, respectively, and $MU_{\mathrm{CDC}}$ is the number of MU calculated by CDC algorithm.

## 3 RESULTS

### 3.1 StereoCalc validation

**FIGURE 2:**
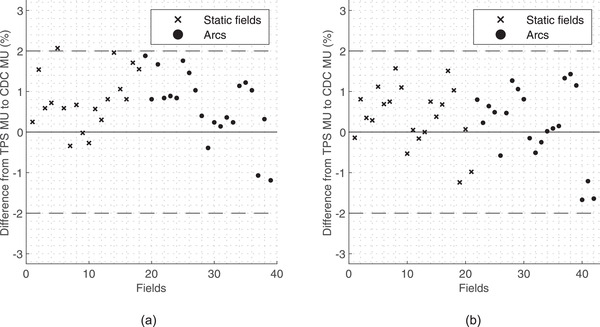
Scatter plot of MU calculation differences between treatment planning systems (TPSs) and StereoCalc for static fields for (a) 6 MV flattening filter‐free (FFF) and (b) 10 MV FFF. (x) static fields and (•) for arcs

The results of the MU calculation without heterogeneity correction, in static fields and arcs for 6 MV FFF and 10 MV FFF energies, are shown in the scatter plots in Figure 2.

**FIGURE 3:**
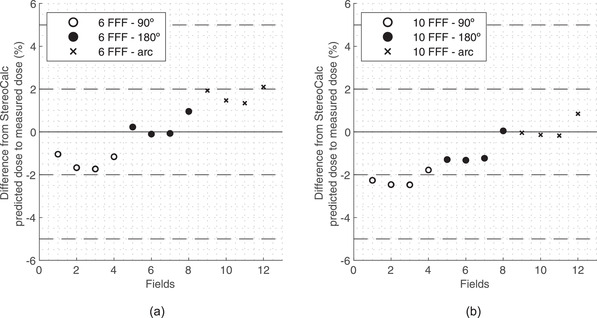
Scatter plot of differences between StereoCalc predicted dose and measured dose for static fields and arcs with (a) 6 MV FFF and (b) 10 MV FFF. (o) for 90° field, (•) for 180° field, and (x) for arc

Figure 3 shows scatter plots with the results of the MU calculation with heterogeneity correction in static fields and arcs for 6 MV FFF and 10 MV FFF energies. The results are divided in three incidences: the 90° lateral beam, the 180° posterior beam, and the arc between 0° and 180° clockwise.

### 3.2 StereoCalc clinical testing

**FIGURE 4:**
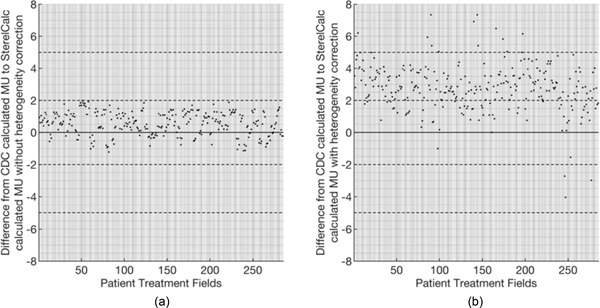
Percentage differences from cone dose calculation (CDC) calculation and the developed algorithm from the 81 patients treated in the TrueBeam STx. (a) Without and with heterogeneities (b)

**FIGURE 5:**
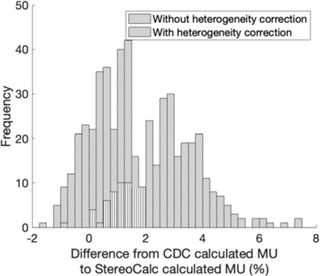
Histogram of the differences from cone dose calculation (CDC) calculation and the developed algorithm from the 81 patients treated in the TrueBeam STx. (forward slash) Without and with heterogeneity corrections (backslash)

The results used to assess the accuracy of the StereoCalc in stereotactic patients, with (a) and without (b) heterogeneities corrections, are presented in Figure 4. For each situation, the mean and the standard deviation are calculated. Figure 5 represents the histogram of the differences between both algorithms with and without heterogeneity correction.

The maximum deviation found for MU calculation without heterogeneity correction was 1.98%. The mean percentual difference of StereoCalc from CDC was 0.78% for all patients. A standard deviation of 0.48% was observed. The obtained results are within the expected, as literature refers the independent verification program is typically not as accurate as the primary calculation (TPS).^1^ For MU calculation with heterogeneity correction, the maximum deviation was 7.34% with a mean percentual difference of 2.86% for all patients and a standard deviation of 1.27%. From the 285 fields of 81 different patients that were analyzed, only 12 fields exceeded the 5% deviation.

## 4 DISCUSSION

### 4.1 StereoCalc validation

StereoCalc validation was performed for MU calculation with and without heterogeneity correction using different methodologies. StereoCalc tends to calculate more MU (dose underestimation) when compared to the CDC, with a maximum difference of 2.07%. All the other values are below 2% difference. For similar calculation algorithms in homogeneous conditions, AAPM TG‐114 states a tolerance of 2% for fields using the same patient or phantom geometry with minimal field‐shaping.^1^ The results are within the expectations because the developed algorithm without heterogeneity correction has a close behavior to the CDC algorithm. However, we expected random results without seeing any tendency to under or overestimate the number of calculated MU. No relationship was found between the difference of both algorithms considering the energy, cone size, and type of beam (static or arc). The difference might be explained by the acquired beam data that were introduced in the StereoCalc configuration. The beam data used for CDC configuration were acquired at the LINAC commissioning and did not follow the TRS‐483 protocol.

MU calculation validation with heterogeneity correction was performed in a QUASAR phantom, with a 90° lateral beam, a 180° posterior beam, and an arc between 0° and 180° clockwise for 6 MV FFF and 10 MV FFF using three cone sizes: 10, 12.5, 15, and 17.5 mm. No comparison was made between StereoCalc and CDC because the latter does not consider heterogeneity corrections. StereoCalc tends to underestimate the dose when compared to measurements, especially at 10 MV FFF. However, in radiosurgery treatments, most of the beams do not cross air cavities before reaching the target volume, and 10 MV is not used as often as 6 MV.^13^ For 6 MV FFF StereoCalc underestimates the dose for the 90° field, overestimates the dose for the arc, and produces mixed results for the 180° field. Depending on the cone size, it under‐ or overestimates the dose.

The largest dose deviation found in this validation was −2.47%. None of the observed differences exceeded the 5% tolerance stated by AAPM TG‐114. This is the uncertainty widely accepted between the difference of the planned dose and the delivered dose, for an effective radiation treatment.^1^

### 4.2 StereoCalc clinical testing

Once phantom validation was completed, the StereoCalc software was applied in 81 radiosurgery patients treated in a Varian TrueBeam STx using cones. Treatment plans were performed in the Varian Cone Planning.

The mean percentage difference between the CDC algorithm and the StereoCalc calculation was 0.78% ± 0.48% without heterogeneities. Considering heterogeneities, the values found were 2.86% ± 1.27%. The standard deviation values for the later situation indicate a higher dispersion of the calculated results.

The percentage difference obtained for plans without heterogeneities is within the range of ±2%, corresponding to the accepted values for similar calculation algorithms in homogeneous conditions. Considering heterogeneities, this percentage difference is slightly higher, reaching as higher as 7.34%. The higher values are presented in patients with target volumes very close to the skull, with certain beam incidences almost parallel to the bone. This type of incidence increases the bone thickness and leads to an increase in the number of MU to compensate for the attenuation. On the other hand, central lesions typically have beam incidences that are perpendicular to the skull, decreasing the amount of bone that the beam crosses on its trajectory. Varian states that the dose error due to bone attenuation is approximately 1.2%^12^; however, the overall attenuation obtained in this study is slightly higher. Only five treatment fields of four different plans have less MU calculated with StereoCalc considering heterogeneities, due to the presence of structures with low radiation attenuation in the beam path, such as the nasal cavity.

An agreement of 2% was found for all tested patients between both algorithms, CDC and StereoCalc. Considering these results, we demonstrate that this can be a useful tool to be used as an independent MU verification.

## 5 CONCLUSIONS

The main objective of this work was the development of an independent MU calculation tool (StereoCalc) for stereotactic treatments with the Varian cone system in a Varian TrueBeam STx. The goal of this independent MU verification is to prevent major errors in dose delivery. This software‐level comparison instead of measurements with an ionization chamber ensures that no additional error is introduced due to the measurements and is less time consuming.

The results obtained in this work show that the MU calculated with StereoCalc software is in good agreement with the values calculated by the CDC in outlined conditions, both in static fields and arcs. We also have improved the software to consider heterogeneity corrections calculations. As expected, and as a major achievement of this work, considerable dose differences were observed when heterogeneities were considered in the calculations.

These differences can be explained due to the presence of high‐density structures like the skull, mainly in treatment plans that have beam incidences close to the bone leading to an increase of the number of MU to compensate the attenuation. This kind of heterogeneity correction gives the ability to the plan executer to weigh and compare different beam incidences, considering the crossing of structures with specific radiation attenuation, improving the accuracy of the treatment plans. Based on these results, appropriate action levels can be determined for the implementation of the independent MU calculator software ‐ StereoCalc. The cause of larger deviations on the established action levels for treatment plans should be further investigated. If necessary, a replanning of the treatment can be performed, or even optimization using phantom measurements can be tried.

As the main conclusion, StereoCalc proved to be a powerful tool that can be used for independent verification in stereotactic treatment with cones and is fully automated without user interference. This type of tool gains more and more importance in a radiotherapy medical physics department, supporting radiation QA, especially in delicate procedures such as precision stereotactic radiosurgery and is recommended in AAPM TG‐114.^1^ This software can also be adapted to other treatment techniques.

The StereoCalc program considers tissue heterogeneities, overcoming the main limitation of the CDC algorithm used in radiosurgery treatment dose calculations. This means that without heterogeneities, based on the results presented in this work, overestimation of calculated dose can occur, leading to patient underdose. These should constitute encouraging results in the direction of the heterogeneity correction introduction in future commercially available SRS planning systems.

## CONFLICT OF INTEREST

The authors declare that there is no conflict of interest that could be perceived as prejudicing the impartiality of the research reported.

## AUTHOR CONTRIBUTION

Guilherme Filipe Pinto Campos, Ana Catarina Souto, and Anabela Gregório Dias conceived the present idea, developed the independent MU calculation software, and performed the respective validations in hospital environment. Joana Borges Lencart and Luís Paulo Teixeira Cunha contributed to the writing of the manuscript providing critical feedback. All authors discussed the results and contributed to the final manuscript.

## Supporting information

Supporting Information
