# Supplementary material for: Development of an independent MU calculation software for radiotherapy treatments with stereotactic cones
Source: J Appl Clin Med Phys. 2022 Feb 15;23(4):e13542. doi: 10.1002/acm2.13542 (PMC8992931; doi:10.1002/acm2.13542)
Supplement: Supplementary file 1 — Supporting Information [file ACM2-23-e13542-s001.docx]

**Development of an independent MU calculation software for radiotherapy treatments with stereotactic cones**

Guilherme Filipe Pinto Campos^a,^^[[1]](#footnote-1)^, Ana Catarina Santos Souto^b,c,^^[[2]](#footnote-2)^, Joana Borges Lencart^b,c^, Luís Paulo Teixeira Cunha^b,c^, Anabela Gregório Dias^b,c^

^a^ Department of Physics and Astronomy, Faculty of Sciences, University of Porto, Porto, Portugal

^b^ Medical Physics, Radiobiology and Radiation Protection Group, IPO Porto Research Centre (CI-IPOP), Portuguese Oncology Institute of Porto (IPO Porto), Porto, Portugal

^c^ Medical Physics Department, Portuguese Oncology Institute of Porto (IPO Porto), Porto, Portugal

Corresponding author e-mail: guilherme.campos.03@gmail.com

**Author Contribution Statement**

Guilherme Filipe Pinto Campos, Ana Catarina Souto and Anabela Gregório Dias conceived the present idea, developed the independent MU calculation software and performed the respective validations in hospital environment. Joana Borges Lencart and Luís Paulo Teixeira Cunha contributed to the writing of the manuscript providing critical feedback. All authors discussed the results and contributed to the final manuscript.

**Acknowledgments**

The authors would like to acknowledge Portuguese Oncology Institute of Porto (IPO Porto) the possibility to carry out this study.

1. Author present address: Departamento de Radioterapia e Oncologia Clínica, Instituto CUF Porto, Porto, Portugal [↑](#footnote-ref-1)
2. Author present address: Mercurius Health S.A., Tagus Park, Núcleo Central Expansão, Porto Salvo, Portugal [↑](#footnote-ref-2)
